# Parental Stress and Child Quality of Life after Pediatric Burn

**DOI:** 10.3390/ebj5020007

**Published:** 2024-03-27

**Authors:** Dinithi Atapattu, Victoria M. Shoesmith, Fiona M. Wood, Lisa J. Martin

**Affiliations:** 1 Fiona Wood Foundation, Burns Unit, Fiona Stanley Hospital, MNH (B) Main Hospital, Level 4, 102-118 Murdoch Drive, Murdoch, WA 6150, Australia; 2 Burn Service of Western Australia, Burns Unit, Fiona Stanley Hospital, MNH (B) Main Hospital, Level 4, 102-118 Murdoch Drive, Murdoch, WA 6150, Australia; 3 Burn Injury Research Unit, University of Western Australia, 35 Stirling Highway, Crawley, WA 6009, Australia

**Keywords:** pediatric, burn, parent, stress, quality of life, post-traumatic growth, coping, psychosocial, outcome, family-centered care

## Abstract

Parents’ emotions after their child’s burn might be influenced by the injury circumstances or demographic characteristics of the patient and family. Parents’ post-traumatic stress symptoms and their child’s distress may interact and affect emotional states. The psychosocial outcomes of parents were measured using the Impact of Event Scale-Revised, the CARe Burn Scale, and the Post-traumatic Growth Inventory-Brief. The psychosocial quality of life outcomes of the pediatric burn patients were measured using the Pediatric Quality of Life Inventory (PedsQL). Regression analysis was used to assess the relationship between patient psychosocial quality of life and the related parent scores. A total of 48 patients and parents participated, with 36 giving full data at 12 months. Parental post-traumatic stress symptoms were initially high, settling by six months, although outliers remained. Parents reported higher IESR scores if their child was female, if they felt helpless at the time of the incident, and if a language other than English was spoken in the home. Parents’ scores of their child’s psychosocial function were similar to their child’s self-scores. Parents who perceived poorer emotional functioning in their child reported higher IESR scores.

## 1. Introduction

In addition to the serious physical impact, recent research discusses the significant psychological distress experienced by pediatric burn patients [1]. Acute burn care is traumatizing for children with multiple hospitalizations, dressing changes, and surgeries regardless of burn severity [2]. Treatment continues after discharge for acute burn management to provide long-term scar management. The journey to recovery can lead to long-term psychological distress and poor quality of life (QoL) in children [3,4], and research shows that pediatric burn patients have higher postburn psychiatric morbidity than children without burn injury, even when the burn is not severe [2]. With the advancement of acute burn management, burn mortality rates are declining, mounting the importance of designing comprehensive aftercare packages for children and their families, addressing both physical and psychological recovery [5,6].

A child suffering a burn is stressful for parents and primary care givers. Parental emotions can be influenced by the circumstances of the burn event or the demographic and social characteristics of the patient and their family [7]. The sudden disruption in their child’s normal life requires parents to have increased involvement in their daily activities, and there can be feelings of guilt or shame and fears of long-term scarring that might affect the body image, mobility, and esteem of the child. All these factors can be emotionally disturbing for parents, leading to difficulties in social functioning, anxiety, depression, and post-traumatic stress disorder (PTSD) [8,9], with mothers being at a greater risk [10].

Parents’ stress can affect their child, and children’s distress is often emotionally difficult for their parents. It is important to understand all potential influences on psychosocial recovery to help pediatric burn patients achieve optimal outcomes. Multiple interdependent factors could cause distress in children and parents to different degrees. Understanding parental subjective appraisal of the injury is crucial to cater to their unique needs [10]. It is important to monitor different cohorts closely and longitudinally. Moreover, little research has been conducted on the psychosocial outcomes of the children and parents at the same time [11].

The aim of this study was to identify factors that could be identified at the time of injury that might predict longer-term post-traumatic stress symptoms in parents of children with non-severe injury. If clinicians can identify parents at risk of experiencing higher levels of post-traumatic stress symptoms early in the postburn journey, they can offer extra support or targeted interventions to reduce the potential of the development of PTSD. Additionally, by improving parental coping and reducing parental post-traumatic stress symptoms, this will benefit the recovery of the patient. We hypothesized that (1) factors identifiable at the time of treatment for acute burn injury could predict parental post-traumatic stress symptoms in the longer term, and (2) that parental post-traumatic stress symptoms would be associated with patient quality of life. Therefore, the aims of the study were to assess the longitudinal progression of psychosocial outcomes and quality of life for parents of pediatric burn patients during the first 12 months postburn, the identification of predictors of longer-term parental post-traumatic stress symptoms that are present or measurable in the acute stages of pediatric burn, and the associations between psychosocial outcomes for parents and patient quality of life.

## 2. Materials and Methods

Ethics approval was granted through the Child and Adolescent Health Service Human Research Ethics Committee at Perth Children’s Hospital (RGS0000003310). The de-identified data presented in this study are available on request from the corresponding author. The data are not publicly available due to HREC privacy requirements. Patients and parents (or primary carers) of patients 2 years or older presenting to Perth Children’s Hospital with an acute burn between 1 July 2020 and 30 September 2022 were invited to participate.

The demographic details collected were age at injury (years), sex (male, female), and postcode of residence. Family data, collected by parent self-report at time of recruitment, included Indigenous status, languages spoken at home other than English, and level of parent education (high school, some tertiary, university). Injury event and clinical data collected from the medical records included location of burn on the body, total body surface area (TBSA%), burn depth (superficial partial, mid-dermal, deep, full thickness), cause of burn (scald, flame, contact, friction, other), type of acute surgery required (split-thickness skin graft, ReCell^TM^, scaffold, a combinations of these, or other), and length of inpatient stay in days (LOS).

### 2.1. Measures

For the parents of the pediatric patients, three self-reported measures were collected.

First, the Impact of Events Scale-Revised (IES-R): The IES-R has 22 items and is used to measure post-traumatic stress symptoms. Parents can self-report this measure, and it takes 5–10 min to complete. This was collected at baseline, at 6 m postburn, and at 12 m postburn. The baseline measure was recorded at 4 weeks, as post-traumatic stress symptoms are defined as the symptoms still present at this timepoint or beyond.

Second, the CARe Burn Scale (CARe) [12]: The CARe Burn Scale assesses the psychosocial effects of the burn scar on quality of life. This is a burn-specific questionnaire which takes about 15 min to complete. Parents score children under 8 years; children 8 years or older score themselves, and parents also score their own quality of life in relation to their child’s burn. This study analyzes the parents’ self-scores. Data for this measure were collected at 6 m and 12 m postburn.

Third, the Post-traumatic Growth Inventory Short Form (PTGI-SF) [13,14]: This measure was used to allow the parents to self-score any indicators of post-traumatic growth (PTG). PTG is not the opposite of post-traumatic stress, but post-traumatic stress can be a precursor of PTG [15]. This is a 10-item measure that has been validated in many areas of trauma, including adult burn patients in the Australian context, but not parents or families of burn patients, to our knowledge. Data for this measure were collected at 6 m and 12 m postburn.

Additionally, data were collected from parents for four potential predictors of parental post-traumatic stress following child injury [16]. These have been previously used in pediatric burn trauma in Western Australia [17] and consist of four dichotomous questions:

1. Did you see the incident (accident) in which your child got hurt?
2. When your child was hurt (or when you first heard it had happened) did you feel really helpless, like you wanted to make it stop but could not?
3. Were you with your child in the ambulance or helicopter on the way to the hospital?
4. Does your child have any behavior problems or problems paying attention?

For pediatric patients, quality of life was measured with The Pediatric Quality of Life Scale Version 4 (PedsQL). This assesses general physical, emotional, social, and school function via a 23–25 item measure. The latter three functions combine to score psychosocial function, with each domain being scored out of one hundred. There are four adaptations for ages from 2 years upwards, with only parents scoring 2- to 4-year-olds and both parents and patients scoring from 5 years upwards. This measure has been validated in various areas of health care, including burns in the Australian setting [18]. This measure determined the lower age limit of the cohort at two years.

### 2.2. Analyses

A descriptive analysis was completed to assess the proportions or percentages of the categorical variables and medians and interquartile ranges for the continuous variables. In addition, median values and interquartile ranges are presented for all questionnaire data. Comparisons of paired medians were completed with the Wilcoxon sign-rank test to assess whether there was a change over time for the 6 m and 12 m Parent CARe scores and PTGI-SF scores and to check whether there were differences between the parent-rated and child-rated PedsQL scores. In addition, the CARe mean scores (and standard deviations) were compared to published norms. Box plots and trajectory plots present the data visually. All analyses were designed to manage non-normal continuous data where appropriate.

Univariate generalized linear models (GLMs) assessed the relationship between the outcome measure of IES-R total score (the dependent variable) and the independent variables (IVs). The IVs included the patient demographic and clinical variables and the predictor variables, as listed above. This was completed for each timepoint at which the IESR was collected (4 weeks, 6 months, 12 months). In addition, the baseline IESR was evaluated to assess whether there was a relationship between that and future IESR scores to assess whether longer-term post-traumatic stress symptoms could be predicted at 4 weeks postburn. The threshold was set at 0.2, and the variables that met this criterion were entered into a multivariate model. A backwards elimination process was used to progressively drop variables with a *p*-value > 0.05 [19]. The relationship between the IES-R scores and parent post-traumatic growth scores, CARe scores, and child PedsQL Psychosocial Function Scores were assessed with Pearson pairwise correlation analyses. All analyses were completed in Stata16 [20].

## 3. Results

### 3.1. Patient and Family Characteristics

Fifty-one patients were recruited to the study; however, two patients withdrew after baseline data collection, and one was lost to follow-up. Regarding the patients, 55% were male (n = 27), and the median age was 7 years (range 2–15). One family identified as Aboriginal, and one family identified as Māori. For area of residence, 75% of families lived in the metropolitan area of Perth (n = 37), with the rest living in regional or rural areas. Parent education levels were reported, and 44% (n = 21) completed university, 35% (n = 17) attended some level of tertiary education, and 21% (n = 10) reported receiving a high school education. There was attrition over time, with 50 patients completing the baseline questionnaires and 36 parents completing questionnaires at 12 months. Age, gender, and TBSA did not statistically differ for those who did not complete the full 12 months compared to those who did.

### 3.2. Clinical Characteristics of the Burn Assessment

Scald accounted for 60.4% (n = 29), contact accounted for 25% (n = 12), and flame accounted for 10.4% (n = 5) of patients, and the remaining two patients had a friction and an electrical burn injury, respectively. The median burn size was 2.25% (range 0.2–12%) total burn surface area (TBSA). Only four patients did not have acute surgery to close the wound; for those who did, 53% received the application of cells only (ReCell^TM^), 12.24% had a split-thickness skin graft (STSG), and the remaining 26.5% had a combination of split-thickness skin graft and cell application. One patient had a negative pressure device over the STSG, and one patient had Biobrane^TM^ applied over the cells.

### 3.3. Event Questions for Parents

Fewer than half the parents witnessed the burn event (~46%, n = 22), and approximately 17% of all parents accompanied their child in the ambulance or helicopter. Approximately three quarters of parents (~73%, n = 35) felt helpless when they witnessed or first heard about the event. One quarter (25%, n = 12) reported that their child had behavior or attention problems. For these four questions, 12.5% (n = 6) reported no predictors of parental post-traumatic stress symptoms, 31.3% (n = 15) reported one predictor, 41.7% (n = 20) reported two predictors, and 14.6% (n = 7) reported three predictors. No parent reported all four predictors.

### 3.4. Impact of Event Scale-Revised

**Figure 1:**
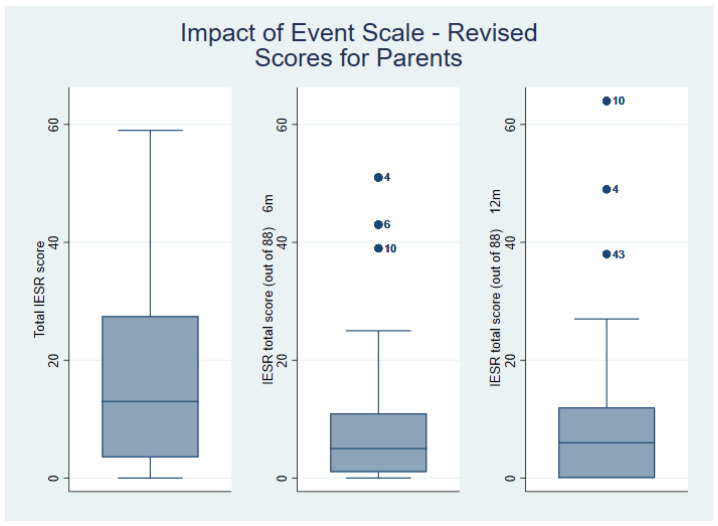
Box Plots for Impact of Event Scale-Revised. The four outliers shown in this figure (#4, #6, #10, #43) had all sustained 2.5–3% scald burns. Two were 5-year-old females, another an 11-year-old female, and the fourth was a 10-year-old male.

The parental baseline post-traumatic stress symptom scores were high but settled by six months for most parents (Table 1, Figure 1). However, some outliers remained at the later timepoints.

### 3.5. Parent CARe Burn Scale

Parental quality of life at 6 months and 12 months postburn was significantly lower than the published scores (mean values reported for comparison) (Table 2). The median paired scores were unchanged between 6 m and 12 m (Table 3). 

### 3.6. Post-Traumatic Growth Scores for Parents

The median parental post-traumatic growth scores were low at 6 m and 12 m, with no significant change over time (Table 3).

### 3.7. Patient-Reported and Parent-Reported Psychosocial Quality of Life Scores

**Figure 2:**
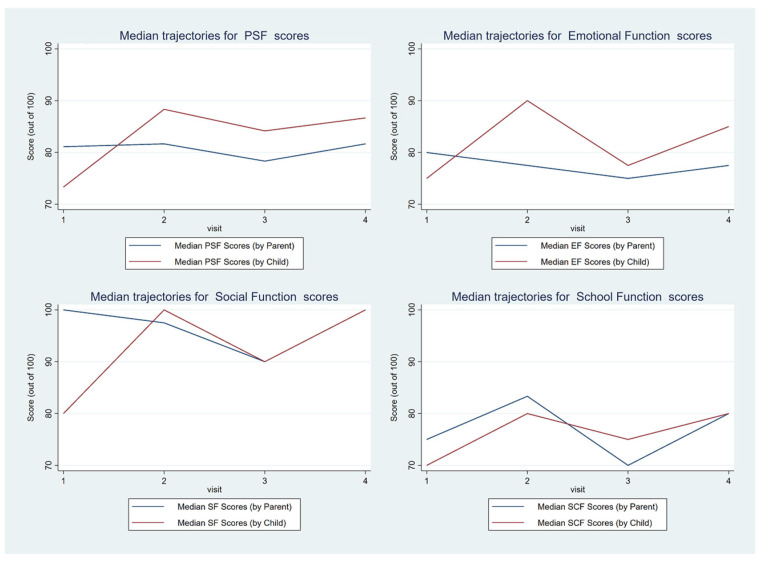
PedsQL trajectories for overall psychosocial function and each subdomain.

Patient quality of life, as measured with the PedsQL, scored high for social function, with the exception of the baseline scores reported by the children. Scores were lower for emotional function and school function, and these two domains lowered the overall psychosocial functions score, which combines the three sub-domains (Figure 2). The parent- and child-reported scores were not different at 3 m, 6 m, and 12 m, reflecting that the parents accurately assess their child’s quality of life in most instances. This only differed for the baseline school function, which will be due to school being missed due to hospital admission, and there is a non-statistically significant effect on social function, which is also likely to be due to hospital admission. Together, these lower the scores for overall psychosocial function for the child-reported scores. Overall, the parents scored similarly to the children for each patient age group.

### 3.8. Regression Analyses

#### 3.8.1. Univariate Analyses for Potential Covariates for Model Assessing Parental Post-Traumatic Stress Symptoms

The association of each independent variable was assessed by generalized linear modeling to assess their effect on IES-R at each timepoint (Supplementary Table S3). These associations determined the variables for inclusion in the initial model.

#### 3.8.2. Multivariate Analysis—Explanatory Factors for Early Parental Post-Traumatic Stress Symptoms

The initial model contained the following covariates: sex, metro residence, parent education, other languages spoken at home, burn type, and predictor 2. After backwards elimination, the baseline model contained the following three covariates: sex, parent education, and predictor 2.

The child being female is associated with a 75% higher likelihood of an increase in IES-R score. In addition, IES-R scores are likely to be 3.9 times greater if the parent felt helpless at the time of the event, and parental education level classified as ‘some tertiary education’ (e.g., TAFE) is associated with a 4.8 times higher likelihood of increased parental post-traumatic stress symptoms (Table 4).

#### 3.8.3. Multivariate Analysis—Explanatory Factors for Longer-Term Parental Post-Traumatic Stress Symptoms

The initial models for each timepoint contained the covariates sex, metro residence, parent education, other languages spoken at home, burn type, and predictors 1, 2, and 3. After backwards elimination, the 6 m and 12 m models contained the three covariates of sex, other language spoken at home, and predictor 2. At 6 months postburn, parents of female children are 2.5 times more likely to report high scores, those who speak other languages at home are 2.2 times more likely to report high scores, and those who feel helpless at the time of the injury are 3.7 times more likely to report high scores. At 12 months postburn, all scores increased and became more statistically significant. Parents of female children are 3.1 times more likely to report high scores, those who speak other languages at home are 4.2 times more likely to report high scores, and those who feel helpless at the time of the injury are 4.6 times more likely to report high scores (Table 4). Importantly, baseline IESR was independently predictive of the IESR scores at 6 m (OR 1.05, 95% CI 1.03, 1.07, *p* < 0.0001) and 12 m (OR 1.05, 95% CI 1.03, 1.07, *p* < 0.0001), and the 6m IESR scores were predictive of the 12 m IESR scores (OR 1.8, 95% CI 1.05, 1.12, *p* < 0.0001).

#### 3.8.4. Pairwise Correlations between Parental Post-Traumatic Stress Symptoms and Parental QoL

The correlations between each individual domain for the CARe quality of life questionnaire are all positive and statistically significant. Parental post-traumatic stress symptoms are inversely related to concerns for the child’s appearance and for social situations with their child. Post-traumatic growth is associated with more post-traumatic stress symptoms, more growth, more appearance concerns, and fewer social situation concerns, and this is consistent at 6 m and 12 m postburn (Table 5 and Table 6).

#### 3.8.5. Pairwise Correlations between Parental Post-Traumatic Stress Symptoms and Child QoL

The parent IES-R scores are significantly inversely related to patient quality of life at 12 months. The patient and parent PedsQL scores are significantly positively correlated (Table 7).

## 4. Discussion

Parent post-traumatic stress symptoms, as measured by the IES-R, were high at 1 month postburn but settled by six months; however, outliers remained. Our first hypothesis was that factors identifiable at the time of treatment for acute burn injury could predict parental post-traumatic stress symptoms in the longer term. We aimed to identify predictors of longer-term parental post-traumatic stress symptoms that were measurable in the acute stages of pediatric burn. The three predictors of parental post-traumatic stress symptoms identified in our cohort were (1) the child being female, (2) languages other than English spoken in the home, and (3) feeling helpless at the time of the incident. Importantly, high parent IES-R scores at 4 weeks postburn were predictive of higher scores at six and 12 months. Thus, this hypothesis was supported.

There are demographic and social characteristics of children which make children and their parents more vulnerable to poor long-term recovery. Female patients are at a higher risk, with long-term anxiety disorders, body image concerns, and depression being reported more frequently in female survivors [21,22]. The finding that parents of daughters have higher post-traumatic stress symptoms levels is supported in the literature [17]. This gender difference postburn extends into education, with females doing less well in school [23], and it extends into adulthood, with females being more vulnerable to poor health-related quality of life after adult burn injury [24] and more at risk of other future health issues such as cancer [25]. Physically, the grip strength of females who were burnt at a younger are lower after their burn compared to males [26]. Socially, the explanatory factor in this cohort of other languages spoken at home might be reflective of other issues. These families had a sound grasp of the English language, even if English was not their first language. These issues could be due to lower socioeconomic status, new immigration or refugee status, or other cultural differences [27,28,29], all of which have been shown to be factors that increase burn risk and vulnerability. Feeling helpless at the time of the injury was a common theme and found to be a risk for higher post-traumatic stress symptoms levels, consistent with the literature [16,17] and related to the finding that parents felt distress because they felt hopeless that they could not fix everything for their child [30]. Helplessness can occur in parents when they are unable to meet their instinct to protect their child from harm, and then feelings of guilt compound this because they feel they could have done more to protect their child [15].

Other factors were not demonstrated to be a risk for longer-term parental post-traumatic stress symptoms in this study. Results from other studies have been mixed, with both pre-school children and adolescents being anxious after burn injury [1]. Burn severity, as measured by TBSA, has been associated with increased post-traumatic stress symptoms in children and parents [31]. However, neither age nor TBSA were identified as a risk factor for higher post-traumatic stress symptom scores in this cohort.

The second hypothesis was that parental post-traumatic stress symptoms would be associated with patient quality of life. We aimed to assess the longitudinal progression of psychosocial outcomes and quality of life for parents of pediatric burn patients during the first 12 months postburn and investigate the relationship between psychosocial outcomes for parents and patient quality of life. The relationships between the two measures supported the hypothesis that as parental post-traumatic stress symptoms rise, child psychosocial quality of life reduces. However, this relationship only reached statistical significance for parent-scored child quality of life at the 12-month timepoint, possibly reflecting how their post-traumatic stress symptoms affect their perceptions of their child.

The relationship between each individual domain for the CARe quality of life questionnaire are positive and highly statistically significant. Questions are rated on a 5-point scale, with some items being scored in reverse. For the overall questionnaire, higher scores indicate better outcomes. However, it appears that the scores for our patient cohort are lower than other values that have been published [12]. This was unexpected; given our cohort profile of non-severe burns, we expected that the scores would be similar to, or better than, published values. The domain about parents’ concerns for the child’s appearance (such as “How concerned are you about how their wounds/scars look overall?”) demonstrated an inverse relationship with the IES-R scores. We expected that if the parent had concerns about the appearance of the child’s scars that they would be more stressed, and thus, this result was not expected. Also, in this cohort, there is an inverse relationship between IESR scores and social situations with the child. The social situation domain consists of questions such as “I feel ok when other people look at my child’s burn wounds/scars”. Thus, the relationship with parental stress and the social situation domain is expected, but the relationship with parental stress and concerns about the appearance of the scar is not expected.

We expected the results of these two associations to potentially support each other. However, this is a cohort of non-severe burn injury, with a median burn size of 2.25%, and scar location in terms of visibility on the body was not collected. Visible and non-visible scars cause different emotional and psychosocial responses, and parents might underestimate the stigmatization experienced by their child [32]. This might influence parental reactions to these two domains. The parent feeling ok about their child’s burn in social situations at 6 months or 12 months after the burn event might indicate acceptance and adequate coping, and this might result in fewer concerns about scar appearance.

Higher post-traumatic growth scores are associated with higher post-traumatic stress symptoms scores. This result was expected as post-traumatic stress symptoms and post-traumatic growth are not opposing constructs and are thought to be synergistically related. Post-traumatic growth after adult burn has been found to be preceded by stress, but stress and growth have been found to occur together, and this has been described as ‘a double track of recovery’ [33]. Post-traumatic growth can be the drop in strength in an ocean of difficulties and can function as a protective coping factor. The post-traumatic growth scores are correlated with the growth domain in the CARe measure, which is an expected outcome. It is also positively associated with appearance concerns and negatively associated with social situation concerns.

The distress for parents after their child has a burn results in memories of the event that are associated with fear, sadness, guilt, and relief [15]. From the moment of injury, through the acute care, then scar care, and beyond, the response of parents can affect the child’s psychological recovery [9,34,35,36]. Our results demonstrate a synergistic relationship between the stress experienced by the parents and the quality of life of the child. When children recognize the guilt and anxiety experienced by their caregivers, it negatively impacts their own mental health [9]. A family-centered approach is required to achieve optimal outcomes for the child.

Clinically, these three factors—female patient, language other than English spoken at home, and feelings of parental helplessness at the time of injury—can be identified with a simple screen. These factors can be regarded as ‘red flags’ for parents who might need extra support over the first 12 months following their child’s burn, and referral for psychological assessment and therapy might benefit these parents. Further research to confirm or repute these findings, and for the testing of appropriate interventions, would be the next step in this field.

## 5. Limitations

The sample size for this study was small, and this study was part of a bigger study that looked at the recovery of children injured via burns in Western Australia. The small sample size might mean that we were unable to detect smaller effect sizes. This cohort of parents is well educated and may not be representative of wider demographics. Caution needs to be applied in extrapolating these results to other pediatric burn populations.

## 6. Conclusions

Parental stress after pediatric burn remained high at 6 and 12 months postburn if the patient was female, if a language other than English was spoken at home, and if the parent felt helpless at the time of the injury. These are all factors that can be identified at the time of burn presentation. The administration of the IES-R, at 4 weeks postburn, to the parents of patients who meet these criteria (at a minimum) could help identify parents at risk of higher post-traumatic stress symptoms levels in the longer term so that extra support can be given. Parental post-traumatic stress symptoms relate to child quality of life, and thus, supporting these parents will provide family-centered care that will help to achieve optimal recovery for the child.

## Figures and Tables

**Table 1:** Impact of Event Scale-Revised scores.

IESR	Median (IQR)	Range
Baseline (4 weeks)	13 (3.5–27.5)	0–59
6 months	5 (1–11)	0–51
12 months	6 (0–12)	0–64

**Table 2:** Comparison of mean CARe scores to published data.

CARe Domain	6-Month Score Mean (SD)	Published Norms 6 m Mean (SD)	*p*-Value
Parent Positive Growth	49.5 (34.73)	61.92 (25.16)	0.0196
Parent concerns for appearance	55.4 (37.83)	82.22 (25.34)	<0.0001 *
Parent Negative Mood Score	54.3 (32.77)	60.06 (12.80)	0.2399
Self Worth/Positive Mood	56.8 (33.58)	70.32 (19.00)	0.0089
Parent Social Situations	57.5 (38.9)	77.74 (28.44)	0.0010 *
Parent Physical Health	50.04 (32.29)	69.56 (24.56)	0.0002 *
Parent Partner Relationships	61.2 (39.68)	72.03 (24.89)	0.0706

* Statistically significant at *p* < 0.05.

**Table 3:** Comparison of median scores between 6 m and 12 m postburn.

CARe Domain	6-Month Score Median (IQR)	12-Month Score Median (IQR)	Wilcoxon Signed-Rank Test *p*-Value
Parent Positive Growth	30 (55–75)	55 (30–86)	0.16
Parent concerns for appearance	61 (10–91)	61 (30–100)	0.07
Parent Negative Mood Score	60 (44–76)	60 (53–85)	0.33
Parent Positive Mood Score	66.5 (51–71)	68 (48–80)	0.51
Parent Social Situations	70 (11–100)	70 (23–100)	0.37
Parent Physical Health	54 (31–71)	54 (31–81)	0.39
Parent Partner Relationships	65 (28–100)	65 (43–100)	0.55
Posttraumatic Growth Score Overall score (out of 50)	19 (9.5–25)	14.5 (6.5–28)	0.22

**Table 4:** Final multivariate model for explanatory factors of IES-R scores at each timepoint.

Dependent Variable	Independent Variable	Odds Ratio	Standard Error	*p*-Value	95% CI
Baseline IESR	Female	1.75	0.498	0.048	1.004, 3.060
	Parent Education	overall test chi^2^ 13.23 (2) *p* = 0.0013			
	some tertiary	4.78	2.509	0.003	1.71, 13.36
	university	2.02	1.049	0.173	0.73, 5.59
	Predictor 2	3.89	1.48	<0.0001	1.85. 8.20
6 month IESR	Female	2.50	0.844	0.006	1.29, 4.85
	Other language	2.23	0.726	0.014	1018, 4.22
	Predictor 2	3.75	1.722	0.004	1.53, 9.23
12 month IESR	Female	3.15	1.290	0.005	1.41, 7.03
	Other language	4.21	1.641	<0.001	1.93, 9.04
	Predictor 2	4.60	20,249	0.002	1.767, 11.99

**Table 5:** Pearson correlations between IES-R scores, CARe scores, and PTG scores at 6 m postburn (* *p* < 0.05).

6-Month Timepoint	6 m IESR	Parent Positive Growth	Parent Concerns for Appearance	Parent Negative Mood Score	Parent Positive Mood Score	Parent Social Situations	Parent Physical Health	Parent Partner Relationships	Post-traumatic Growth Score
6-month IESR	1								
Parent Positive Growth	0.21	1							
Parent concerns for appearance	−0.39 *	0.52 *	1						
Parent Negative Mood Score	−0.14	0.75 *	0.72 *	1					
Parent Positive Mood Score	−0.15	0.75 *	0.72 *	0.93 *	1				
Parent Social Situations	−0.57 *	0.51 *	0.86 *	0.74 *	0.81 *	1			
Parent Physical Health	−0.16	0.67 *	0.63 *	0.84 *	0.89 *	0.69 *	1		
Parent Partner Relationships	−0.14	0.69 *	0.65 *	0.88 *	0.85 *	0.66 *	0.76 *	1	
Post-traumatic Growth Score	0.49 *	0.46 *	−0.49 *	0.22	0.03	−0.51 *	0.04	0.22	1

**Table 6:** Pearson correlations between IES-R scores, CARe scores, and PTG scores at 12 m postburn (* *p* < 0.05).

12-Month Timepoint	12 m IESR	Parent Positive Growth	Parent Concerns for Appearance	Parent Negative Mood Score	Parent Positive Mood Score	Parent Social Situations	Parent Physical Health	Parent Partner Relationships	Post-traumatic Growth Score
12-month IESR	1								
Parent Positive Growth	−0.01	1							
Parent concerns for appearance	−0.34 *	0.72 *	1						
Parent Negative Mood Score	−0.27	0.84 *	0.81 *	1					
Parent Positive Mood Score	−0.15	0.84 *	0.82 *	0.96 *	1				
Parent Social Situations	−0.33 *	0.67 *	0.89 *	0.83 *	0.87 *	1			
Parent Physical Health	−0.25	0.79 *	0.74 *	0.88 *	0.92 *	0.79 *	1		
Parent Partner Relationships	0.15	0.84 *	0.75 *	0.89 *	0.89 *	0.71 *	0.86 *	1	
Post-traumatic Growth Score	0.38 *	0.49 *	−0.34 *	0.0425	0.14	−0.28	0.22	0.35 *	1

**Table 7:** Pearson correlations between IES-R scores and PedsQL scores.

Baseline		IESR	PedsQL PSF (Parent-scored)	PedsQL PSF (Child-scored)
	IESR	1		
	PedsQL PSF (Parent-scored)	−0.1281	1	
	PedsQL PSF (Child-scored)	−0.1788	0.5068 * p = 0.0019	1
6 m	IESR	1		
	PedsQL PSF (Parent-scored)	−0.2618	1	
	PedsQL PSF (Child-scored)	−0.2739	0.6986 * p = 0.0001	1
12 m	IESR	1		
	PedsQL PSF (Parent-scored)	−0.4615 * p = 0.0060	1	
	PedsQL PSF (Child-scored)	−0.1726	0.4198 * p = 0.0411	1

* Statistically significant at *p* < 0.05.

## Data Availability

The data are not publicly available due to HREC privacy requirements. The de-identified data presented in this study are available on request from the corresponding author. Applicants will require appropriate ethics permissions.

## Supplementary Materials

The following supporting information can be downloaded at: https://www.mdpi.com/article/10.3390/ebj5020007/s1. Table S1: Comparison of parent and child PedsQL scores for overall psychosocial function and each subdomain; Table S2: Parent and Child Comparison of PedsQL PSF scores by age group; Table S3: Univariate analysis of IES-R scores and each potential covariate at each time-point.
