# Supplementary material for: Parental Stress and Child Quality of Life after Pediatric Burn
Source: Eur Burn J. 2024 Mar 27;5(2):77–89. doi: 10.3390/ebj5020007 (PMC11545290; doi:10.3390/ebj5020007)
Supplement: Supplementary file 1 [file ebj-05-00007-s001.zip › ebj-2688491-supplementary.pdf]

Supplementary Tables S1-3

Table S1 Comparison of parent and child PedsQL scores for overall psychosocial function and each subdomain

	Visit	Child Score Median (IQR)	Parent Score Median (IQR)	Wilcoxon signed-rank test p-value
PSF overall score	Baseline	73.3 (58.3-88.3)	81.1 (72.2-91.9)	0.0259
	3m	88.3 (56.7-96.7)	81.7 (68.3-96.7)	0.5
	6m	84.2 (63.3-91.7)	78.3 (70.5-91.7)	0.6
	12m	86.7 (70.0-96.7)	81,7 (66.7-96.7)	0.5
Emotional Function	Baseline	75.0 (55.0-90.0)	80.0 (65.0-90.0)	0.3
	3m	90.0 (60.0-100)	77.5 (65.0-95.0)	0.2
	6m	77.5 (60.0-90.0)	75.0 (55.0-95.0)	0.6
	12m	85.0 (70.0-100)	77.5 (62.5-95)	0.3
Social Function	Baseline	80.0 (65.0-100)	100 (75.0-100)	0.0541
	3m	100 (75.0-100)	97.5 (75.0-100)	0.5
	6m	90.0 (70.0-100)	90.0 (75.0-100)	0.4
	12m	100 (75.0-100)	100 (75.0-100)	0.2
School Function	Baseline	70.0 (60.0-80.0)	75.0 (65.8-90.0)	0.0145
	3m	80.0 (60.0-90.0)	83.3 (65.0-95.0)	0.9
	6m	75.0 (60.0-80.0)	70.0 (65.0-90.0)	0.9
	12m	80.0 (70.0-90.0)	80.0 (65.0-100)	0.7

Table S2 Parent and Child Comparison of PedsQL PSF scores by age group

Psychosocial Function PedsQL	Child Score Median (IQR)	Parent Score Median (IQR)	Wilcoxon signed-rank test p-value
Toddler (2-4y)	Not applicable	87.8 (76.1-96.7)	-
Young Child (5-7)	78.3 (63.3-90)	81.7 (66.7-91.7)	0.3
Child (8-12)	88.3 (65-95)	81.7 (68.3-93.3)	0.9
Teen (13+)	86.7 (73.3-90)	80.83 (70-90)	0.8

Table S3 Univariate analysis of IES-R scores and each potential covariate at each time-point

Dependent Variable	Independent Variable	Odds Ratio	Standard Error	P-Value	95% CI
Baseline IESR	Female	1.93	0.543	0.019*	1.11, 3.33
	Age	1.01	0.338	0.7	0.95, 1.08
	Metro	1.77	0.640	0.116**	0.86, 3.59
	Parent Education	overall test chi² 9.64(2) p=0.0081**			
	- some tertiary	8.87	6.649	0.004	2.04, 38.54
	- university	16.75	12.159	<0.0001	1.04, 69.49

	Other Language	0.31	.0445	0.4	0.68, 2.55
	Scald	1.59	0.450	0.099**	0.92, 2.77
	Contact	0.52	0.209	0.102**	0.23, 1.14
	Flame	0.422	0.186	0.050*	0.18, 1.00
	TBSA	0.99	0.046	0.9	0.91, 1.09
	Predictor 1	0.86	0.259	0.6	0.48, 1.55
	Predictor 2	2.38	0.947	0.057**	0.98, 5.13
	Predictor 3	0.92	0.331	0.8	0.46, 1.86
	Predictor 4	0.93	0.318	0.8	0.74, 1.82
6m IESR	Female	2.49	0.938	0.016*	1.19, 5.21
	Age	1.02	0.582	0.7	0.92, 1.17
	Metro	1.34	0.551	0.5	0.59, 3.00
	Parent Education	overall test chi^2 2.18(2) p=0.337			
	- some tertiary	1.64	0.831	0.333	0.60, 4.43
	- university	1.99	0.953	0.149	0.78, 5.09
	Other Language	2.49	1.121	0.043*	1.03, 6.02
	Scald	2.29	0.851	0.26*	1.11, 4.75
	Contact	0.51	0.209	0.102**	0.23, 1.14
	Flame	0.60	0.221	0.166**	0.29, 1.24
	TBSA	1.07	0.117	0.6	0.86, 0.32
	Predictor 1	0.89	0.406	0.8	0.36, 2.17
	Predictor 2	4.49	2.392	0.005*	1.58, 12.75
	Predictor 3	0.36	0.188	0.050*	0.13, 1.00
	Predictor 4	1.28	0.834	0.7	0.36, 4.59
12m IESR	Female	2.41	1.207	0.079**	0.90, 6.43
	Age	1.04	0.066	0.6	0.92, 1.17
	Metro	2.67	1.557	0.093**	0.85, 8.38
	Other Language	3.41	1.599	0.009*	1.36, 8.55
	Parent Education	overall test chi^2 15.20(2) p=0.0005			
	- some tertiary	8.87	6.649	0.004	2.04, 38.54
	- university	16.75	12.159	<0.0001	1.04, 69.49
	Scald	3.76	1.75	0.004*	1.51, 9.36
	Contact	0.35	0.156	0.019*	0.15, 0.84
	Flame	-	-	-	-
	TBSA	1.24	0.223	0.240	0.87, 1.76
	Predictor 1	0.54	0.241	0.165**	0.22, 1.29
	Predictor 2	6.05	3.192	0.001*	2.15, 17.02
	Predictor 3	0.07	0.057	0.001*	0.02, 0.33
	Predictor 4	2.05	1.177	0.212	0.66, 6.32
